# Genetic variants of ANP and cardiometabolic protection: from populations to novel therapeutics

**DOI:** 10.1186/2050-6511-14-S1-O7

**Published:** 2013-08-29

**Authors:** Valentina Cannone, Paul M McKie, Angelo Baldassare Cefalu, Davide Noto, Giovanni Cavera, Michele Pagano, Michelangelo Sapienza, Timothy M Olson, Denise M Heublein, Christopher G Scott, Kent R Bailey, Maurizio Averna, John C Burnett

**Affiliations:** 1 Cardiorenal Research Laboratory, Mayo Clinic, Rochester, MN, USA; 2 Department of Internal Medicine and Medical Specialities, University of Palerma, Italy; 3 Az. Osp. Ospedali Riuniti Villa Sofia-Cervello, Palermo, Italy; 4 Ingrassia hospital, Palermo, Italy; 5 Cardiovascular Genetics Research Laboratory, Divisions of Cardiovascular Diseases and Pediatric Cardiology, Mayo Clinic, Rochester, MN, USA; 6 Division of Biostatics, Mayo Clinic, Rochester, MN, USA

## Background

The cardiac hormone atrial natriuretic peptide (ANP) induces natriuresis, vasodilation and inhibits aldosterone through the activation of the guanylyl cyclase A receptor (GC-A) and the second messenger cGMP. ANP possesses also metabolic properties enhancing lipolysis and release of the adipokine adiponectin. Previous studies in general populations reported that the minor G allele of the ANP genetic variant rs5068 is associated with increased circulating levels of ANP and B-type natriuretic peptide, lower blood pressure (BP), and reduced risk of hypertension. We recently reported that in the general population from Olmsted County, MN, USA the G allele of rs5068 is associated not only with increased levels of ANP and lower BP but also with lower BMI, prevalence of obesity and metabolic syndrome.

To advance our understanding of the phenotype associated with rs5068 we analyzed a community-based cohort from Sicily, Italy. Our second aim was to advance a potential therapeutics for cardiometabolic disease employing a novel long-acting ANP, MANP whose biological actions were defined in canines and in a rat model of metabolic syndrome.

## Results

In a Sicilian cohort (n=812) genotype frequencies of rs5068 are AA: 93.5%, AG: 6.4%, and GG: 0.1%. After adjusting for age and gender, the minor G allele is associated with lower BMI (estimate (SE): -1.7 (0.8) kg/m^2^, p=0.04) and prevalence of metabolic syndrome (p=0.02), obesity tends to be less prevalent (p=0.07). Independently of age, gender and BMI, the minor=allele is associated with lower systolic blood pressure (estimate (SE): -6.0 (2.5) mmHg, p=0.02) and prevalence of hypertension (odds ratio: 0.41; 95% CI 0.20 to 0.83; p=0.01). In canines, subcutaneous administration of MANP (10 ug/kg) resulted in a ten-fold greater activation of plasma cGMP (AUC: 5095+-1365 vs 676+-211 pmol/ml/min, p<0.05) and a sustained reduction in BP (p<0.05) when compared to ANP. In a rat model of metabolic syndrome, intravenous administration of MANP (0.13ug/kg/min) significantly decreased BP when compared to baseline (171 vs 186 mmHg, p=0.0024) and increased plasma levels of adiponectin (37 vs 27 ng/ml, p=0.03).

## Conclusion

The association between the minor allele of rs5068 and a “favorable” cardiometabolic phenotype is now replicated in a Mediterranean population. In canines, the novel natriuretic peptide MANP has a sustained cGMP-mediated blood pressure lowering action. In a rodent model of metabolic syndrome, MANP decreases blood pressure and mediates increase of circulating adiponectin. Our findings support further studies to evaluate MANP as a possible therapeutic for cardiometabolic disease.

